# Retrospective cohort study investigating association between precancerous gastric lesions and colorectal neoplasm risk

**DOI:** 10.3389/fonc.2024.1320020

**Published:** 2024-02-20

**Authors:** Hui Pan, Yu-Long Zhang, Chao-Ying Fang, Yu-Dai Chen, Li-Ping He, Xiao-Ling Zheng, Xiaowen Li

**Affiliations:** ^1^ Gastrointestinal Endoscopy Center, Fujian Shengli Clinical Medical College, Fujian Medical University, Fuzhou, Fujian, China; ^2^ Department of Gynecology, Fujian Maternity and Child Health Hospital, Fuzhou, Fujian, China; ^3^ Gastrointestinal Endoscopy Center, Fujian Provincial Hospital South Branch, Fuzhou, Fujian, China

**Keywords:** helicobacter pylori, intestinal metaplasia, atrophic gastritis, colorectal adenoma, serrated lesions

## Abstract

**Background:**

Colorectal cancer (CRC) is considered the most prevalent synchronous malignancy in patients with gastric cancer. This large retrospective study aims to clarify correlations between gastric histopathology stages and risks of specific colorectal neoplasms, to optimize screening and reduce preventable CRC.

**Methods:**

Clinical data of 36,708 patients undergoing gastroscopy and colonoscopy from 2005-2022 were retrospectively analyzed. Correlations between gastric and colorectal histopathology were assessed by multivariate analysis. Outcomes of interest included non-adenomatous polyps (NAP), conventional adenomas (CAs), serrated polyps (SPs), and CRC. Statistical analysis used R version 4.0.4.

**Results:**

Older age (≥50 years) and *Helicobacter pylori* infection (HPI) were associated with increased risks of conventional adenomas (CAs), serrated polyps (SPs), non-adenomatous polyps (NAP), and colorectal cancer (CRC). Moderate to severe intestinal metaplasia specifically increased risks of NAP and CAs by 1.17-fold (95% CI 1.05-1.3) and 1.19-fold (95% CI 1.09-1.31), respectively. For CRC risk, low-grade intraepithelial neoplasia increased risk by 1.41-fold (95% CI 1.08-1.84), while high-grade intraepithelial neoplasia (OR 3.76, 95% CI 2.25-6.29) and gastric cancer (OR 4.81, 95% CI 3.25-7.09) showed strong associations. More advanced gastric pathology was correlated with progressively higher risks of CRC.

**Conclusion:**

Precancerous gastric conditions are associated with increased colorectal neoplasm risk. Our findings can inform screening guidelines to target high-risk subgroups, advancing colorectal cancer prevention and reducing disease burden.

## Introduction

Colorectal cancer (CRC) stands as the third most diagnosed cancer globally and the second leading cause of cancer mortality (1). The incidence and mortality of CRC have been rising rapidly in China, with over 400,000 new cases and 195,600 deaths reported in 2016 (2), making it the second most common cancer diagnosis and fourth leading cause of cancer mortality nationwide. The surge in CRC incidence and mortality in China, underscores the urgent need for effective screening and preventive measures.

While population endoscopic screening is crucial for early detection and removal of premalignant polyps (3), the rates of colonoscopy screening in China lag behind those of upper endoscopy. This discrepancy becomes especially pertinent when clinicians, prompted by findings in gastrointestinal (GI) screening, must make decisions about the necessity of a follow-up colonoscopy, known for its superior detection of colorectal neoplasms.

Recent studies, primarily conducted in Western populations, suggest that certain upper GI pathologies identified during gastroscopy may indicate a higher concurrent or subsequent risk for colorectal neoplasms (4–6). Notably, gastric ulcers, Helicobacter pylori (*H. pylori*) infection, and chronic atrophic gastritis have been associated with an increased prevalence of colorectal adenomas and cancer, possibly due to downstream effects on the GI environment (7–9).

The connection between upper gastrointestinal diseases, such as gastric polyps (10, 11), *H. pylori* infection (12), and reflux esophagitis (13), and elevated colon neoplasm risk has been established in prior evidence. Although the exact mechanisms underlying gastrointestinal diseases remain unclear, lipopolysaccharide (LPS) may play a key role. As a component of Gram-negative bacteria, LPS promotes gastrointestinal diseases through multiple pathways. In the stomach, it activates TGF-beta and Wnt signaling, inducing epithelial-mesenchymal transition and immunotherapy resistance, thereby facilitating gastric cancer progression (14). In the intestines, LPS incites inflammation and tumorigenesis by modulating epithelial signaling cascades (15). Through these diverse mechanisms of stimulating cancer-associated signals in both the gastric and intestinal epithelia, LPS serves as an intermediary factor linking chronic inflammation to carcinoma in gastrointestinal diseases. Besides, this association is potentially linked to the impairment of the gastric acid barrier (16) and the use of proton pump inhibitors (17, 18). However, uncertainties persist regarding whether gastric histopathology, reflecting the pathological state and acid secretion, is directly related to colon neoplasms (19, 20).

A significant Shanghai study involving 5,986 patients shed light on the potential correlation between certain gastric histopathologies—such as atrophic gastritis, intestinal metaplasia, and gastric polyps—and the predisposition to advanced colorectal polyps, as opposed to non-advanced polyps or colorectal cancer (20). This observation underscores the potential value of gastric histopathology in predicting high-risk colon neoplasms specifically within the Chinese population.

However, to validate and generalize these findings, larger population studies across diverse regions are imperative. Such studies would confirm the links between gastric pathology, acid secretion, and susceptibility to colon neoplasms, thereby refining screening practices. Consequently, large-scale analyses leveraging national endoscopy data are essential to elucidate region-specific gastric-colorectal connections in China and optimize screening protocols. This comprehensive retrospective study aims to clarify correlations between gastric histopathology stages and risks of specific colorectal neoplasms, to optimize screening and reduce preventable CRC.

## Methods

### Study design and data selection

This retrospective cross-sectional study was approved by the Academic Ethics Committee of Fujian Provincial Hospital (K2022-01-019) prior to conducting research. Data were retrieved from a scientific research big data platform at Fujian Provincial Hospital, comprising patient demographics, pathological reports, and endoscopic reports (including *H. pylori* status and specimen requisitions). Patients who underwent endoscopy at the Digestive Endoscopy Center from Jan 01, 2005, to Jan 01, 2022 were included. Given the retrospective nature of the study, informed consent was not required.

**Figure 1:**
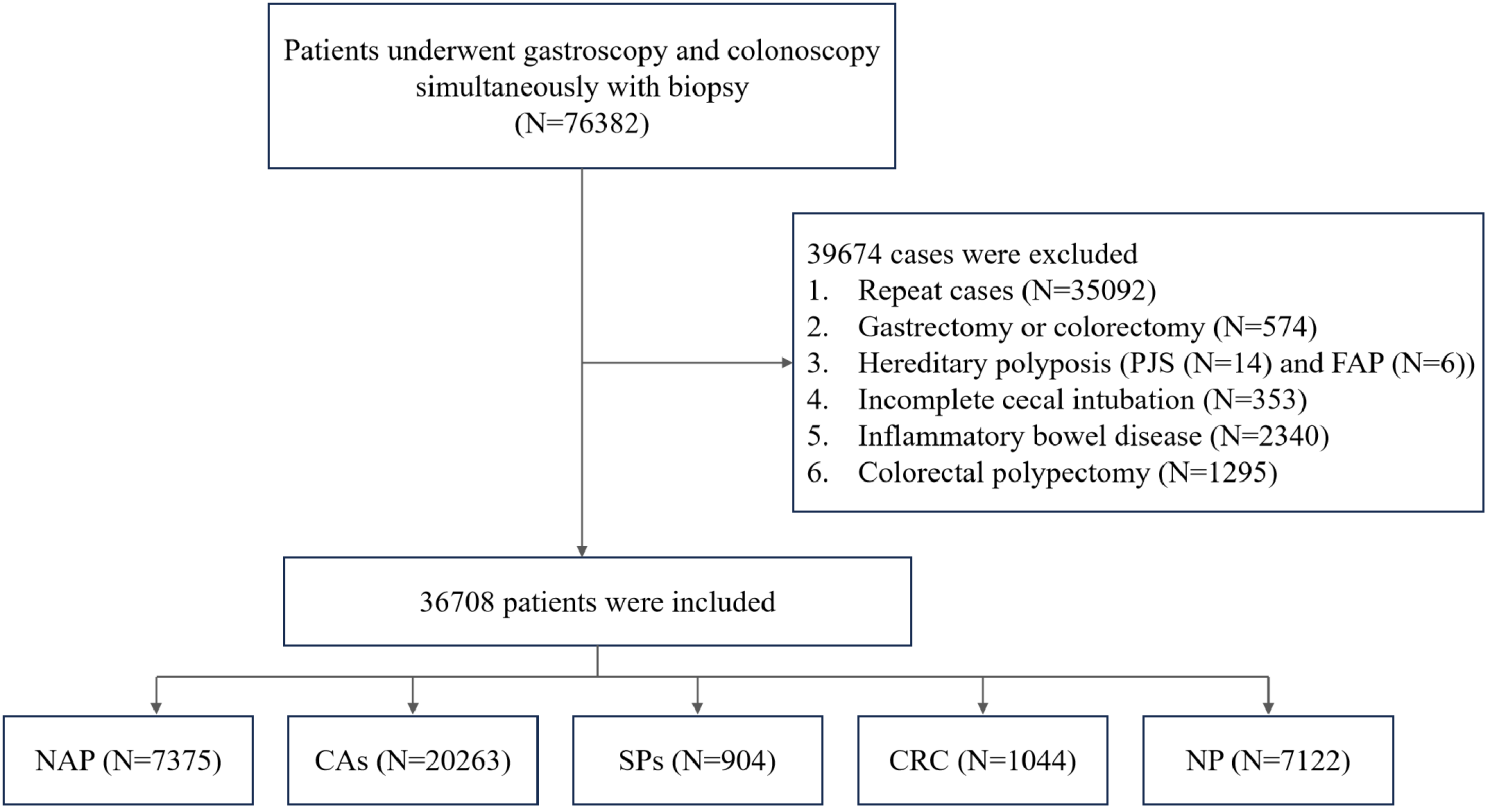
Flow chart of the study design. PJS, Peutz–Jeghers syndrome; FAP, familial adenomatous polyposis; NAP, non-adenomatous polyps; CAs, conventional adenomas; SPs, serrated polyps; CRC, colorectal cancer; NP, no polyps.

The study enrolled patients who underwent simultaneous gastroscopy and colonoscopy with tissue biopsy within 2 months at Fujian Provincial Hospital campuses. Exclusion criteria were as follows: history of gastrectomy or colorectomy, history of colorectal polypectomy, inflammatory bowel disease, hereditary polyposis syndromes like Peutz–Jeghers or familial adenomatous polyposis, and incomplete cecal intubation. Data from the index visit were used, excluding repeat cases. Inclusion and exclusion criteria are detailed in the provided flowchart (**Figure 1**).

### Diagnosis of gastric and colorectal lesions

All gastric histopathological diagnoses were based on the guidelines from the updated Sydney System and Japanese Gastric Cancer Association classification (21). Pathological samples were collected into 10% buffered formalin, embedded in paraffin, sectioned at 2μm, and were sent to the Pathology Department for subsequent staining with hematoxylin and eosin (H&E) and evaluation. Gastric precursor lesions were categorized as atrophic gastritis (AG), intestinal metaplasia (IM - mild, moderate, severe), and dysplasia [low-grade intraepithelial neoplasia (LGIN), high-grade intraepithelial neoplasia (HGIN), gastric cancer (GC)] based on the pathology reports. *H. pylori* infection (HPI) status was assessed via rapid urease test of gastric antrum biopsies.

Colorectal lesions were classified into 5 subgroups per pathology reports which based on WHO classification of tumors of digestive system (22): (1) colorectal cancer (CRC); (2) serrated polyps (SPs) including traditional serrated adenomas and sessile serrated lesions; (3) conventional adenomas (CAs) with tubular/villous components, regardless of dysplasia grade; (4) non-adenomatous polyps (NAP) like hyperplastic or inflammatory polyps; (5) no polyp (NP) control group.

### Statistical analysis

R language (version 4.0.4) software was used for data analysis and visualization. The histogram distribution or Q-Q plot was utilized to assess the normality of the variables. The mean ± standard deviation (SD) was used to express normally distributed continuous data, while skewed continuous variables were characterized using the median and interquartile range (IQR). The categorical variables were displayed as frequencies expressed as percentages. An analysis was conducted to compare continuous variables between groups using independent samples. The choice between Student’s t-test or Mann-Whitney U-test was based on the normality of the distribution. Categorical data were compared using either the chi-square test or Fisher’s exact test, depending on the appropriateness (**Supplementary Figures 1**–**3**). Univariate and multivariate logistic regression (adjusted for gender, age, H. pylori, and atrophy) was used to determine the relationship between gastric precursor lesions and colorectal conditions, including colorectal polyps and CRC. The results were reported as an adjusted odds ratio (OR) with a corresponding 95% confidence interval (CI). For all analyses, a P-value < 0.05 was statistically significant.

## Results

### Baseline characteristics of various colorectal conditions.

A total of 36,708 patients were included in this study and divided into 5 groups based on colonoscopy and biopsy findings: CRC (n=1,044); CAs (n=20,263); SPs (n=904); NAP (n=7,375); and NP (n=7,122). The proportion of males was significantly higher in the CRC (60.9%, 636/1,044), CAs (62.4%, 12,647/20,263), SPs (58.2%, 526/904), and NAP (61.2%, 4,512/7,375) groups compared to the NP control group (55%) (P < 0.001).

The mean age of patients with CRC (63.2 ± 11.6 years) was significantly higher than those with CAs (56.4 ± 10.5 years), SPs (56.1 ± 11.7 years), NAP (52.7 ± 10.9 years) and NP (53.4 ± 11.7 years) (P < 0.001).

The prevalence of gastric mucosal abnormalities was higher in the CRC, CAs, SPs and NAP groups compared to the NP control group. Specifically, the prevalence of gastric intestinal metaplasia (IM) was 41.3% in CRC (20.3% mild, 21% moderate/severe), 41.8% in CAs (21.6% mild, 20.2% moderate/severe), 38.5% in SPs (21.1% mild, 17.4% moderate/severe), 39.3% in NAP (21.2% mild, 18.1% moderate/severe), and 36.5% in NP (19.8% mild, 16.7% moderate/severe) (P<0.001). The prevalence of gastric dysplasia and cancer was 14.5% in CRC, 7.9% in CAs, 9% in SPs, 6.3% in NAP, and 6.1% in NP (P<0.001), with higher rates of low- and high-grade dysplasia and gastric cancer in the CRC group compared to the other groups (**Table 1**).

**Table 1:** Baseline characteristics of various colorectal conditions.

Variables	Total (n = 36708)	no polyps (NP) (n = 7122)	non adenomatous polyp (NAP) (n = 7375)	conventional adenomas (CAs) (n = 20263)	serrated polyps (SPs) (n = 904)	colorectal cancer (CRC) (n = 1044)	p
Gender, n (%)							< 0.001
Male	22236 (60.6)	3915 (55)	4512 (61.2)	12647 (62.4)	526 (58.2)	636 (60.9)	
Female	14472 (39.4)	3207 (45)	2863 (38.8)	7616 (37.6)	378 (41.8)	408 (39.1)	
Age, (Mean ± SD)	55.3 ± 11.1	53.4 ± 11.7	52.7 ± 10.9	56.4 ± 10.5	56.1 ± 11.7	63.2 ± 11.6	< 0.001
H. pylori (+), n (%)	21142 (57.6)	3923 (55.1)	4232 (57.4)	11766 (58.1)	573 (63.4)	648 (62.1)	< 0.001
Atrophic gastritis, n (%)	22040 (60.0)	4081 (57.3)	4348 (59)	12446 (61.4)	533 (59)	632 (60.5)	< 0.001
IM, n (%)							< 0.001
Mild	7755 (21.1)	1411 (19.8)	1564 (21.2)	4377 (21.6)	191 (21.1)	212 (20.3)	
Mod-Severe	7000 (19.1)	1186 (16.7)	1338 (18.1)	4100 (20.2)	157 (17.4)	219 (21)	
Neoplasm, n (%)							< 0.001
LGIN	2153 (5.9)	337 (4.7)	399 (5.4)	1274 (6.3)	66 (7.3)	77 (7.4)	
HGIN	254 (0.7)	36 (0.5)	33 (0.4)	154 (0.8)	5 (0.6)	26 (2.5)	
GC	320 (0.9)	63 (0.9)	37 (0.5)	162 (0.8)	10 (1.1)	48 (4.6)	

Data are presented as number (%) or mean ± SD. A chi-square test was used for categorical variables, and a t-test was used for continuous variables. IM, intestinal metaplasia; Mod-Severe, Moderate-severe; LGIN, low-grade intraepithelial neoplasia; HGIN, high-grade intraepithelial neoplasia; GC, gastric cancer.

### Association between gastric conditions with NAP

**Figure 2:**
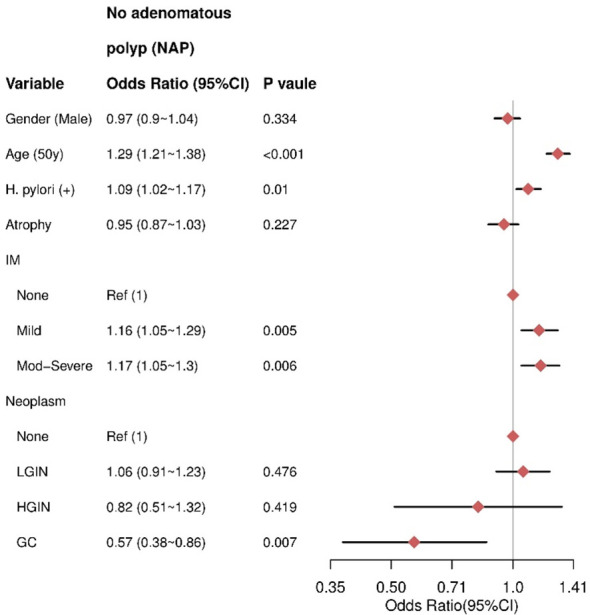
The result of multivariate logistic regression for the NAP group. IM, intestinal metaplasia; LGIN, low-grade intraepithelial neoplasia; HGIN, high-grade intraepithelial neoplasia; GC, gastric cancer.

Multivariate analysis revealed that older age (≥50 years) (OR= 1.29, 95% CI = 1.21–1.38) and HPI (OR = 1.09, 95% CI = 1.02–1.17) were independent risk factors for NAP. IM was also an independent risk factor for NAP, with moderate to severe IM having a higher odds ratio (OR = 1.17, 95% CI = 1.05–1.3) compared to mild IM (OR = 1.16, 95% CI = 1.05–1.29). In contrast, male sex (P = 0.334) and atrophic gastritis (AG) (P = 0.227) were not significantly associated with NAP. Additionally, there was no significant association between LGIN (P = 0.476) or HGIN (P = 0.419) and NAP (**Figure 2**).

### Association between gastric conditions with CAs

**Figure 3:**
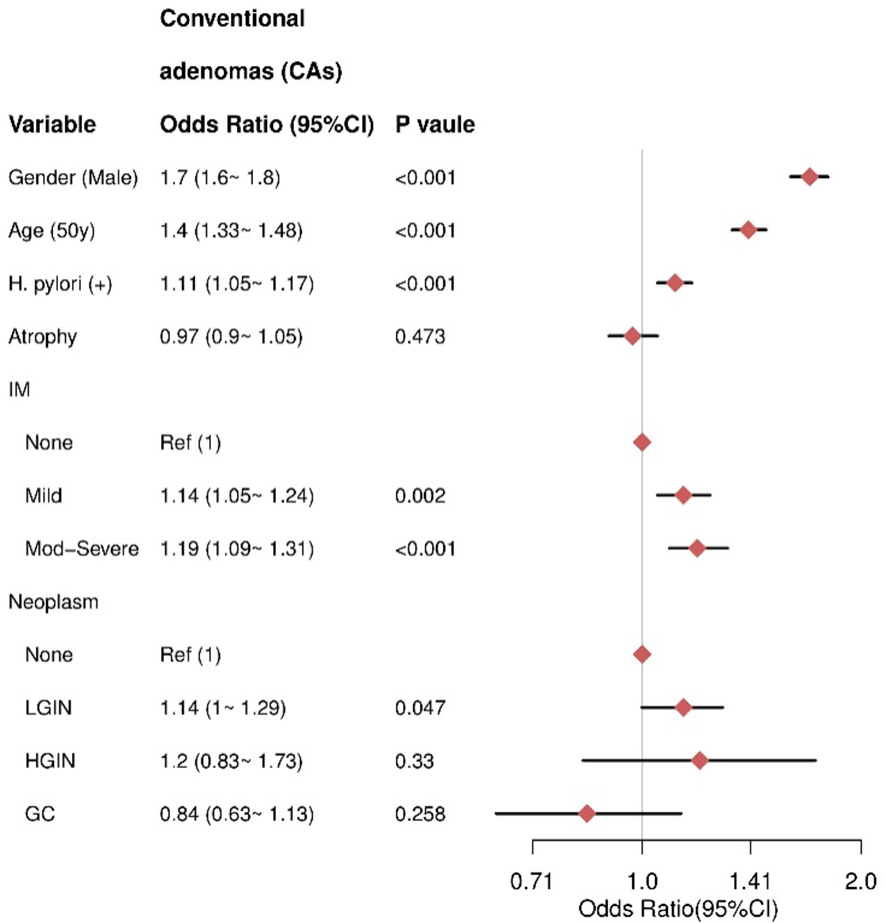
The result of multivariate logistic regression for the CAs group. IM, intestinal metaplasia; LGIN, low-grade intraepithelial neoplasia; HGIN, high-grade intraepithelial neoplasia; GC, gastric cancer.

Multivariate analysis revealed that male sex (OR= 1.7, 95% CI = 1.6–1.8), older age (≥50 years) (OR= 1.4, 95% CI =1.33–1.48), and HPI (OR = 1.11, 95% CI = 1.05–1.17) were independent risk factors for CAs. IM was also a risk factor, with moderate to severe IM associated with higher risk (OR = 1.19, 95% CI = 1.09–1.31) compared to mild IM (OR = 1.14, 95% CI = 1.05–1.24). There was no significant association between AG and CAs (P = 0.473). For gastric neoplasms, HGIN (P = 0.33) and GC (P = 0.258) were not significantly associated with CAs (**Figure 3**).

### Association between gastric conditions with SPs

**Figure 4:**
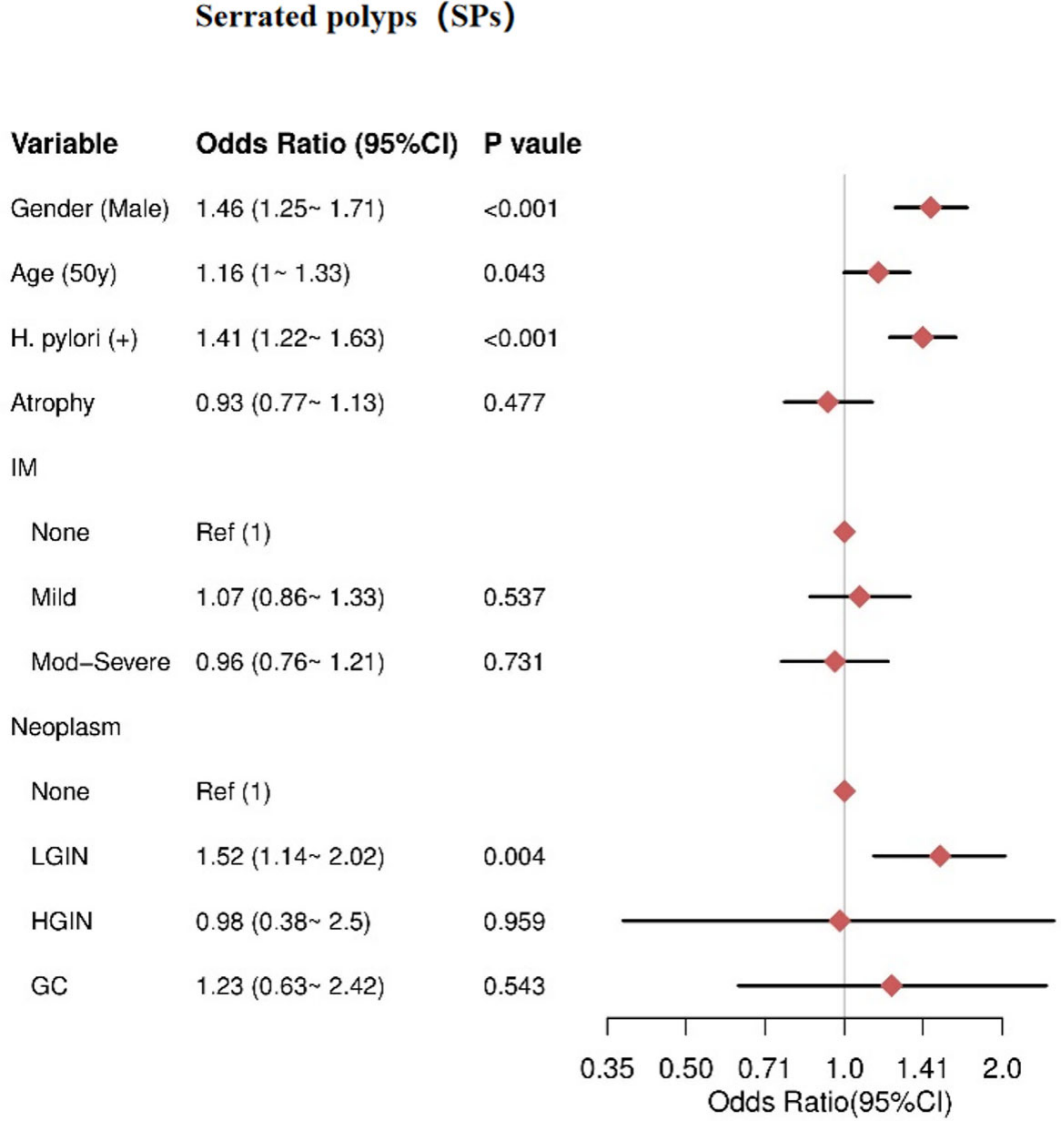
The result of multivariate logistic regression for the SPs group. IM, intestinal metaplasia; LGIN, low-grade intraepithelial neoplasia; HGIN, high-grade intraepithelial neoplasia; GC, gastric cancer.

Multivariate analysis revealed that male sex (OR= 1.46, 95% CI = 1.25–1.71), HPI (OR = 1.41, 95% CI = 1.22–1.63), and older age (≥50 years) (OR = 1.16, 95% CI = 1–1.33) were independent risk factors for SPs. There was no significant association between AG and SPs (P = 0.477). Additionally, IM severity was not significantly associated with SPs, including mild IM (P = 0.537) and moderate-severe IM (P = 0.731). For gastric neoplasms, only LGIN showed an increased risk for SPs (OR = 1.52, 95% CI = 1.14–2.02), while HGIN (P = 0.959) and GC (P = 0.543) were not significantly associated with SPs (**Figure 4**).

### Association between gastric conditions with CRC

**Figure 5:**
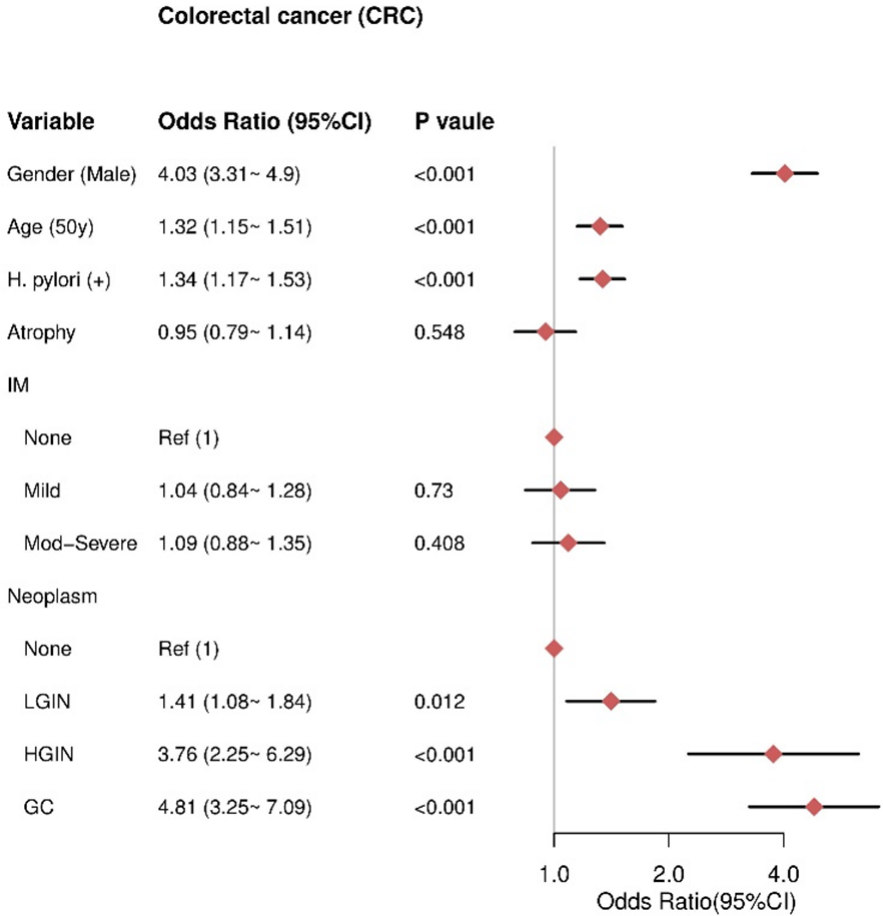
The result of multivariate logistic regression for the CRC group. IM, intestinal metaplasia; LGIN, low-grade intraepithelial neoplasia; HGIN, high-grade intraepithelial neoplasia; GC, gastric cancer.

Multivariate analysis revealed that male sex (OR= 4.03, 95% CI = 3.31–4.9), older age (≥50 years) (OR= 1.32, 95% CI = 1.15–1.51), and HPI (OR = 1.34, 95% CI = 1.17–1.53) were independent risk factors for CRC. Gastric neoplasms were also independent risk factors for CRC, including LGIN (OR = 1.41, 95% CI = 1.08–1.84), HGIN (OR = 3.76, 95% CI = 2.25–6.29), and GC (OR = 4.81, 95% CI = 3.25–7.09), with higher risks associated with more advanced neoplasms. There was no significant association between AG and CRC (P = 0.548) or IM severity and CRC, including mild IM (P = 0.73) and moderate-severe IM (P = 0.408) (**Figure 5**).

## Discussion

This comprehensive retrospective study, involving 36,708 patients who underwent gastroscopy and colonoscopy between 2005 and 2022, presents compelling evidence that precancerous gastric conditions may increase the risk of colorectal neoplasms. Multivariate analysis was employed to assess correlations between various gastric histopathological findings and different subtypes of colorectal neoplasms, including non-adenomatous polyps, conventional adenomas, serrated polyps, and colorectal cancer.

Our study’s results demonstrate that advanced age (≥50 years) and *Helicobacter pylori* infection (HPI) significantly elevate the risk across all assessed colorectal neoplasm subtypes, including conventional adenomas, serrated polyps, non-adenomatous polyps, and colorectal cancer (CRC). These findings align with prior evidence suggesting that chronic inflammation induced by *H. pylori* predisposes individuals to colorectal tumorigenesis. It is noteworthy that these risk factors share significant potential connections and similarities (23–25). HPI triggers chronic gastritis, which can progress to mucosal atrophy, intestinal metaplasia, and reduced gastric acid production (16), further heightening the risk of intestinal diseases.

Additionally, our study revealed an independent association between moderate to severe intestinal metaplasia (IM) and non-adenomatous polyps (NAP), implying that IM may serve as a risk factor for NAP. Unlike previous research that considered atrophy and IM together (20), our investigation differentiated between them. We found that atrophy alone was not linked to an increased risk of NAP, but moderate to severe IM was significantly associated with a higher risk of NAP development. This suggests that the severity of IM, rather than the mere presence of both atrophy and IM, may be a crucial factor affecting the association with NAP.

Similar results were observed for conventional adenomas (CAs). Gastric atrophy did not elevate the risk of CAs, whereas the presence of IM emerged as an independent risk factor for CAs development. Specifically, we found no significant association between gastric atrophy and the occurrence of CAs. In contrast, the presence of IM was linked to an increased risk of CAs.

However, our study did not establish a clear association between low-grade or high-grade gastric epithelial dysplasia (LGIN and HGIN) and NAP or CAs. In our study, only LGIN was identified as a significant risk factor for serrated polyps (SPs) (OR = 1.52, 95% CI = 1.14-2.02). No clear associations were observed between HGIN (P = 0.959) or gastric cancer (GC) (P = 0.543) and SPs. The heightened risk of SPs associated with LGIN but not advanced neoplasia suggests that early gastric lesions may play a particularly influential role in promoting serrated pathway colon carcinogenesis. These differential effects of gastric precancerous stages imply that specific interactions, rather than overall atrophic changes, may exert an influence on this subtype of colorectal neoplasms.

It is noteworthy that LGIN increases the risk of CRC, with an odds ratio (OR) of 1.41 (95% CI 1.08-1.84). HGIN exhibits an even stronger association with the risk of CRC, with an OR of 3.76 (95% CI 2.25-6.29). Gastric cancer itself demonstrates the highest correlation with CRC, with an OR of 4.81 (95% CI 3.25-7.09). This risk stratification based on the degree of gastric pathology aligns with the Correa cascade model of gastric carcinogenesis, supporting an underlying field effect that influences the risk of both gastric and colorectal neoplasia.

Previous research has documented bidirectional synchronous or metachronous occurrences of colorectal and gastric cancers, with 0.7-1.3% of gastric cancer patients subsequently developing colorectal cancer (26, 27), and approximately 5% of colorectal cancer patients experiencing gastric cancer development (28, 29). Prevalence studies have also highlighted an increased incidence of colorectal neoplasms among gastric cancer patients when compared to control groups. For instance, Lee et al. (7) reported a prevalence of colorectal neoplasms in 35.8% of gastric cancer patients, contrasting with 17.9% in the control group. Similarly, Park et al. (30) found a higher prevalence of colorectal adenoma (39.6% vs. 28.6%) and cancer (3.5% vs. 1.3%) in gastric cancer patients versus the control group. Collectively, these findings consistently demonstrate an epidemiological link between gastric and colorectal neoplasms, underscoring the potential for shared carcinogenic processes.

Several studies have identified specific pathways contributing to the development and metastasis of both gastric and colorectal cancers, providing deeper insights into the connections between gastrointestinal tumors (31, 32). The association between premalignant gastric lesions and colorectal neoplasms also suggests the necessity of updating screening recommendations for high-risk patient subgroups.

Additionally, while the significant Shanghai study involving 5,986 patients offered valuable insights into potential correlations between specific gastric histopathologies—such as atrophic gastritis, intestinal metaplasia, and gastric polyps—and the predisposition to advanced colorectal polyps (20), it did not differentiate between non-advanced polyps and colorectal cancer. Our research in Fujian, boasting a larger sample size, not only corroborates and expands upon the findings from the Shanghai study but also delves deeper into nuanced associations between gastric pathology and different stages of colorectal neoplasms.

The divergence in our findings may be attributed to diverse population characteristics in Fujian, shedding light on region-specific variations in the gastric-colorectal connection. These disparities underscore the importance of conducting large-scale studies in specific regions to tailor screening guidelines effectively. Our study provides nuanced insights that can significantly influence screening strategies, enabling the targeted identification and prevention of colorectal neoplasms in high-risk subgroups within the Fujian population.

It is important to acknowledge some limitations in our study. The cross-sectional design of our research prevented us from assessing temporality and establishing causation. Prospective cohort studies that track outcomes over time are needed to confirm predictive relationships between gastric abnormalities and subsequent colorectal neoplasm development. Furthermore, our analysis only adjusted for a limited set of confounding factors including gender, age, H. pylori, and atrophy, which may have introduced residual biases. Expanding adjustments for socioeconomic status, lifestyle factors, and comorbidities could improve isolation of the association with gastric lesions. Additionally, expanding the diversity of our study sample could enhance the generalizability of our results across different populations. However, despite these limitations, our findings strongly support the existence of associations between premalignant stomach lesions and the development of colorectal tumors, suggesting potential utility of this association in guiding screening approaches. Our results open new directions for future research with the potential to significantly impact the prevention and burden of CRC.

In conclusion, this study shows that certain precancerous gastric conditions are associated with a higher risk of colorectal neoplasms. Our results can be used to optimize secondary prevention initiatives targeting high-risk subgroups for enhanced screening. Further research that focuses on the connections between gastric and colorectal diseases may significantly contribute to curbing the global burden of preventable CRC.

## Data availability statement

The original contributions presented in the study are included in the article/**Supplementary Material**. Further inquiries can be directed to the corresponding author.

## Author contributions

HP: Writing – original draft, Writing – review & editing. YZ: Writing – original draft, Writing – review & editing. CF: Data curation, Writing – original draft. YC: Data curation, Writing – original draft. LH: Writing – original draft. XZ: Project administration, Writing – review & editing. XL: Visualization, Writing – review & editing.

## Supplementary material

Supplementary Figure 1 The age distribution histogram in the database.

Supplementary Figure 2 The distribution of age (box plot) across different tumor types. Median value (white text) was present in the plot. no polyps (NP) (n = 7122), non adenomatous polyp (NAP) (n = 7375), conventional adenomas (CAs) (n = 20263), serrated polyps (SPs) (n = 904, colorectal cancer (CRC) (n = 1044).

Supplementary Figure 3 Bar chart of categorical variables in the database.
