# Supplementary figures and images for: Retrospective cohort study investigating association between precancerous gastric lesions and colorectal neoplasm risk
Source: Front Oncol. 2024 Feb 20;14:1320020. doi: 10.3389/fonc.2024.1320020 (PMC10914248; doi:10.3389/fonc.2024.1320020)

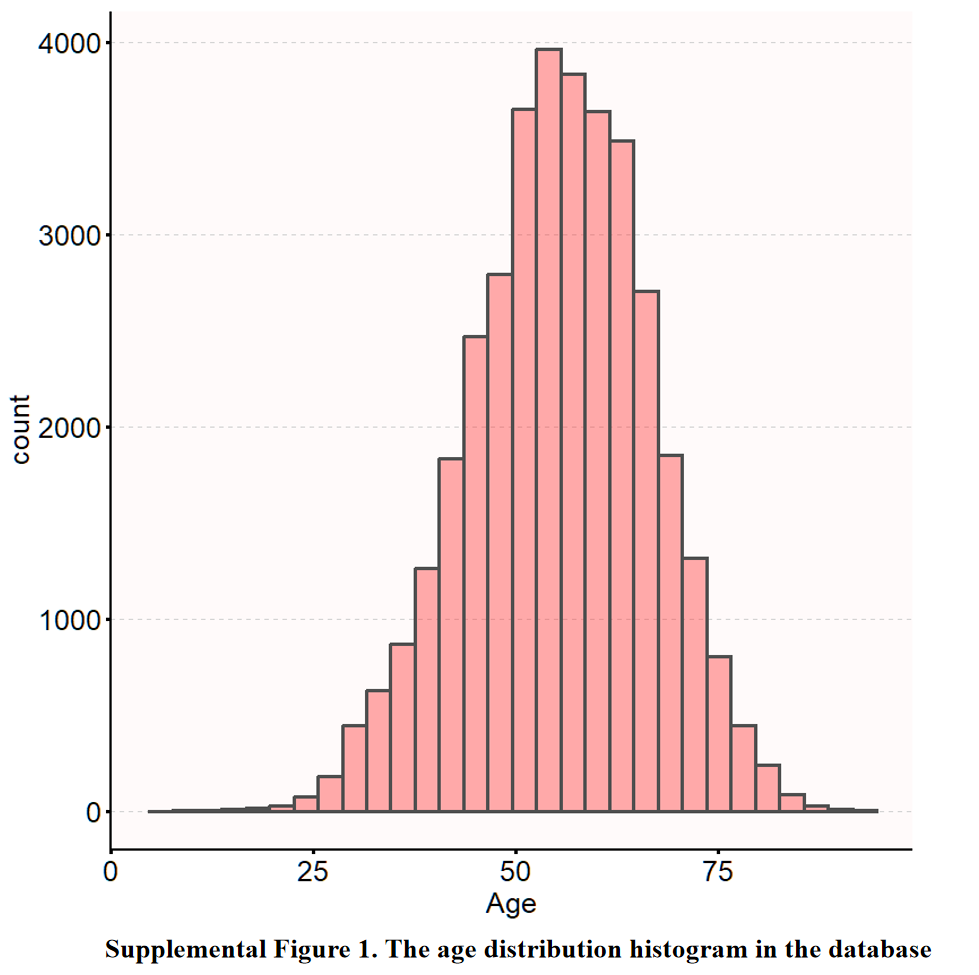

Supplement: Supplementary Figure 1 — The age distribution histogram in the database. [file Image_1.tif]

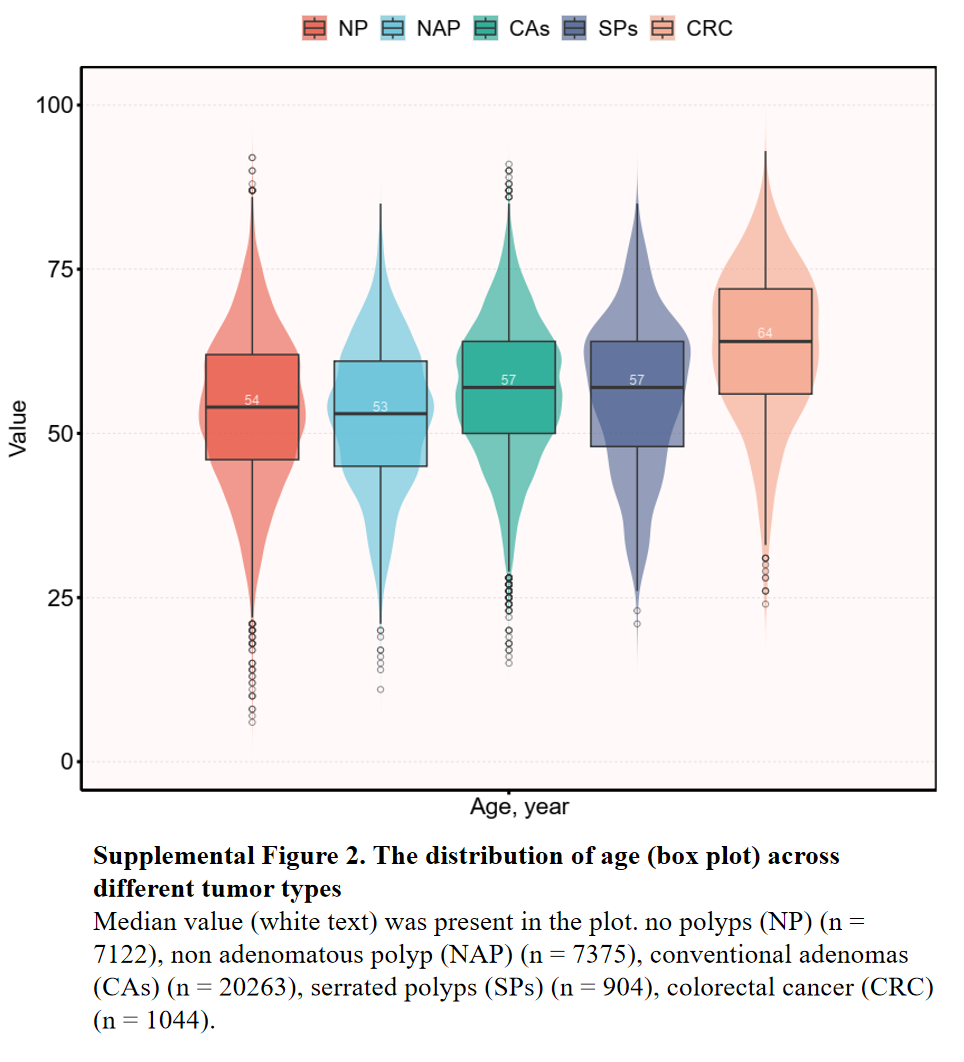

Supplement: Supplementary Figure 2 — The distribution of age (box plot) across different tumor types. Median value (white text) was present in the plot. no polyps (NP) (n = 7122), non adenomatous polyp (NAP) (n = 7375), conventional adenomas (CAs) (n = 20263), serrated polyps (SPs) (n = 904, colorectal cancer (CRC) (n = 1044). [file Image_2.tif]

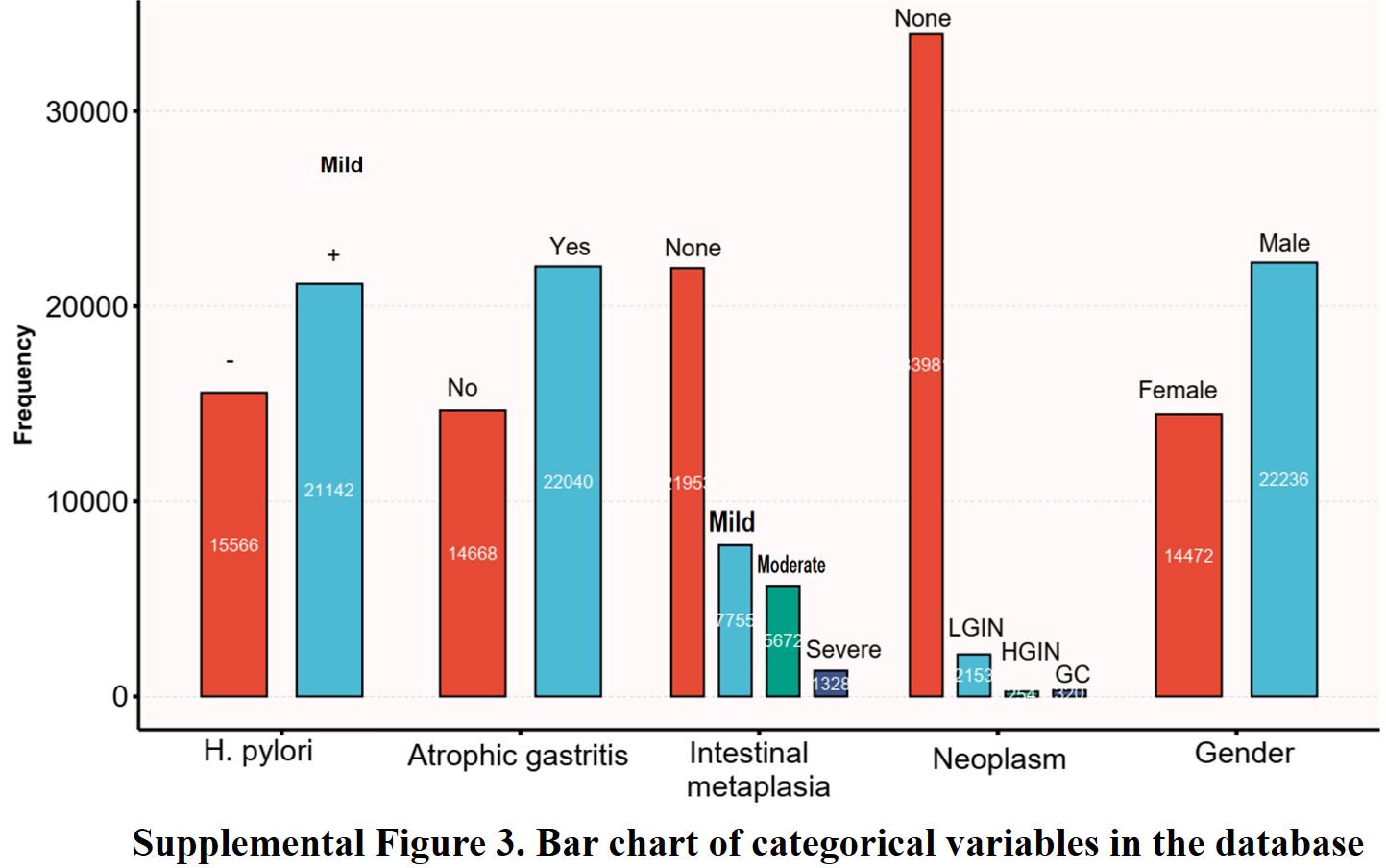

Supplement: Supplementary Figure 3 — Bar chart of categorical variables in the database. [file Image_3.tif]
