# Supplementary material for: Retrospective cohort study investigating association between precancerous gastric lesions and colorectal neoplasm risk
Source: Front Oncol. 2024 Feb 20;14:1320020. doi: 10.3389/fonc.2024.1320020 (PMC10914248; doi:10.3389/fonc.2024.1320020)
Supplement: Supplementary file 4 [file Table_1.doc]

**Supplemental Table 1. The result of univariate logistic regression for the** **various colorectal conditions**

| Variables | No polyps (NP) | Non adenomatous polyp (NAP) | | Conventional adenomas (CAs) | | Serrated polyps (SPs) | | Colorectal cancer (CRC) | |
| --- | --- | --- | --- | --- | --- | --- | --- | --- | --- |
|  | *Ref.* | *OR(95%CI)* | *P value* | *OR(95%CI)* | *P value* | *OR(95%CI)* | *P value* | *OR(95%CI)* | *P value* |
| Gender (Male) | *Ref.* | 1.29 (1.21~1.38) | <0.001 | 1.4 (1.33~ 1.48) | <0.001 | 1.16 (1.01~ 1.33) | 0.042 | 1.32 (1.15~ 1.51) | <0.001 |
| Age (50y) | *Ref.* | 0.96 (0.9~1.03) | 0.225 | 1.69 (1.6~ 1.79) | <0.001 | 1.46 (1.25~ 1.7) | <0.001 | 4.17 (3.43~ 5.06) | <0.001 |
| H. pylori (+) | *Ref.* | 1.1 (1.03~1.17) | 0.005 | 1.13 (1.07~ 1.19) | <0.001 | 1.41 (1.22~ 1.63) | <0.001 | 1.33 (1.17~ 1.52) | <0.001 |
| Atrophy | *Ref.* | 1.07 (1~1.14) | 0.044 | 1.19 (1.12~ 1.25) | <0.001 | 1.07 (0.93~ 1.23) | 0.34 | 1.14 (1~ 1.31) | 0.048 |
| IM |  |  |  |  |  |  |  |  |  |
| None | *Ref.* | 1(Ref) |  | 1(Ref) |  | 1(Ref) |  | 1(Ref) |  |
| Mild | *Ref.* | 1.12 (1.03~1.22) | 0.007 | 1.19 (1.11~ 1.28) | <0.001 | 1.1 (0.92~ 1.31) | 0.277 | 1.11 (0.94~ 1.31) | 0.225 |
| Mod-Severe | *Ref.* | 1.14 (1.04~1.25) | 0.003 | 1.33 (1.23~ 1.43) | <0.001 | 1.08 (0.89~ 1.3) | 0.437 | 1.36 (1.15~ 1.61) | <0.001 |
| Neoplasm |  |  |  |  |  |  |  |  |  |
| None | *Ref.* | 1(Ref) |  | 1(Ref) |  | 1(Ref) |  | 1(Ref) |  |
| LGIN | *Ref.* | 1.15 (0.99~1.33) | 0.072 | 1.35 (1.2~ 1.53) | <0.001 | 1.59 (1.21~ 2.09) | 0.001 | 1.71 (1.32~ 2.21) | <0.001 |
| HGIN | *Ref.* | 0.89 (0.55~1.43) | 0.622 | 1.53 (1.06~ 2.21) | 0.022 | 1.13 (0.44~ 2.89) | 0.795 | 5.41 (3.25~ 9) | <0.001 |
| GC | *Ref.* | 0.57 (0.38~0.85) | 0.007 | 0.92 (0.69~ 1.23) | 0.579 | 1.29 (0.66~ 2.52) | 0.46 | 5.7 (3.89~ 8.36) | <0.001 |

IM: intestinal metaplasia; Mod-Severe: Moderate-severe; LGIN: low-grade intraepithelial neoplasia; HGIN: high-grade intraepithelial neoplasia; GC: gastric cancer.
